# Comparative effectiveness and cost-effectiveness of antiretroviral therapy and pre-exposure prophylaxis for HIV prevention in South Africa

**DOI:** 10.1186/1741-7015-12-46

**Published:** 2014-03-17

**Authors:** Sabina S Alistar, Philip M Grant, Eran Bendavid

**Affiliations:** 1 Department of Management Science and Engineering, Stanford University, Stanford, CA, USA; 2 Division of Infectious Diseases, Department of Medicine, Stanford University, Stanford, CA, USA; 3 Division of General Medical Disciplines, Department of Medicine, Stanford University, Stanford, CA, USA; 4 Center for Health Policy and the Center for Primary Care and Outcomes Research, Stanford University, Stanford, CA, USA

**Keywords:** ART, Cost-effectiveness analysis, HIV epidemic, Oral pre-exposure prophylaxis

## Abstract

**Background:**

Antiretroviral therapy (ART) and oral pre-exposure prophylaxis (PrEP) are effective in reducing HIV transmission in heterosexual adults. The epidemiologic impact and cost-effectiveness of combined prevention approaches in resource-limited settings remain unclear.

**Methods:**

We develop a dynamic mathematical model of the HIV epidemic in South Africa’s adult population. We assume ART reduces HIV transmission by 95% and PrEP by 60%. We model two ART strategies: scaling up access for those with CD4 counts ≤ 350 cells/μL (Guidelines) and for all identified HIV-infected individuals (Universal). PrEP strategies include use in the general population (General) and in high-risk individuals (Focused). We consider strategies where ART, PrEP, or both are scaled up to 100% of remaining eligible individuals yearly. We measure infections averted, quality-adjusted life-years (QALYs) gained and incremental cost-effectiveness ratios over 20 years.

**Results:**

Scaling up ART to 50% of eligible individuals averts 1,513,000 infections over 20 years (Guidelines) and 3,591,000 infections (Universal). Universal ART is the most cost-effective strategy at any scale ($160-$220/QALY versus comparable scale Guidelines ART expansion). General PrEP is costly and provides limited benefits beyond ART scale-up ($7,680/QALY to add 100% PrEP to 50% Universal ART). Cost-effectiveness of General PrEP becomes less favorable when ART is widely given ($12,640/QALY gained when added to 100% Universal ART). If feasible, Focused PrEP is cost saving or highly cost effective versus status quo and when added to ART strategies.

**Conclusions:**

Expanded ART coverage to individuals in early disease stages may be more cost-effective than current guidelines. PrEP can be cost-saving if delivered to individuals at increased risk of infection.

## Background

Despite recent successes in reducing the global burden of HIV, an estimated 2.3 million people were newly infected in 2012, with 1.6 million in sub-Saharan Africa [1,2]. The expansion of combination antiretroviral therapy (ART) for treatment of HIV-infected individuals has been closely linked with mortality reductions in many sub-Saharan African countries [3]. Expansion of ART was accomplished in large part with development assistance programs funded by donor governments and philanthropic organizations [4]. However, assistance for HIV has leveled since 2010, and declined in 2012, increasing the need to consider the value of investments in directing scarce resources for HIV treatment and prevention [5].

While many HIV programs in sub-Saharan Africa invested heavily in expanding ART coverage, scientific advances in recent years have resulted in four new HIV prevention interventions: male circumcision, topical microbicides, oral pre-exposure prophylaxis (PrEP), and ART for prevention [6-10]. Among these, PrEP and ART have generated particular interest because of their efficacy, safety, and, coming on the heels of the expansion in ART for HIV treatment, possibility of large-scale implementation.

ART for prevention, previously supported by observational studies, gained widespread legitimacy with the release of the HPTN052 trial results, which demonstrated that early use of ART in sero-discordant couples reduced HIV transmission by 96% [9]. This finding supported the notion that epidemic control can be achieved by expanding treatment to all those infected, regardless of disease stage. Indeed, real-world examples outside of clinical trials support the effectiveness of expanded ART in preventing HIV infections [11]. By comparison, World Health Organization guidelines recommended initiating treatment when the CD4^+^ T-cell count is at or below 350 cells/μL [12]. More recently, the guidelines have been expanded to include a broader population, although country-specific guidelines lag behind [13,14]. Even in South Africa, where national guidelines support ART for more individuals than any resource-limited country, it is unclear when ART initiation would expand to those with higher CD4 cell counts.

Two recent clinical trials have shown that uninfected heterosexual individuals receiving an oral daily fixed-dose combination of tenofovir disoproxil fumarate and emtricitabine have a 63% to 73% reduced chance of acquiring HIV [15,16]. Another trial demonstrated similar effectiveness among men who have sex with men [8]. However, two trials conducted among African women had study arms stopped for futility, with strong evidence suggesting that the lack of efficacy was due to poor adherence to study medication [17,18]. Despite these concerns, oral PrEP is currently considered for policy implementation in developing countries [19,20]. A prior analysis considered use of PrEP only in heterosexual sero-discordant couples, a situation when the uninfected partner is at high risk of acquiring HIV, and found it can be a potentially cost-saving intervention [21]. Other studies analyzed a limited number of scale-up scenarios and had shorter time horizons [22]. One study estimating the cost-effectiveness of ART for prevention (without PrEP) uses a long time frame [23]. We studied the population health outcomes and cost-effectiveness of implementing expanded ART coverage and oral PrEP in a setting with a heavy HIV burden.

## Methods

### Overview

We built a dynamic compartmental model to represent the HIV epidemic in South Africa’s adult population. Key model parameters are shown in Table 1. The model follows individuals’ health based on their infection status, HIV disease stage, and ART or PrEP use. Full details of the model structure are provided in Additional file 1 – Model details. Our main outcome measures include infections averted, costs, and quality-adjusted life years (QALYs) for each strategy, and the relative cost-effectiveness of strategies.

**Table 1:** Key model parameters with source and ranges (epidemic and behavioral characteristics, intervention scale and effectiveness, health and intervention costs)

**Parameter**	**Value and source**	**Range**
**Prevalence**		
HIV prevalence	17.9% [24]	17.2% to 18.3%
**ART**		
Initial access to ART	40% [24]	35% to 45%
ART quit rate (annual)	0.02 Est	0.00 to 0.20
Sexual transmission reduction	95% [9]	50.0% to 99.0%
**PrEP**		
PrEP quit rate (annual)	0.05 Est	0.00 to 0.20
Sexual acquisition reduction	60% [15,16]	10% to 90%
**Sexual behavior**		
Number of sexual partners per year - general	1.5 [25], Est	1 to 2
Number of sexual partners per year - high risk	4 Est	3 to 5
Condom usage rate - general	25% [25], Est	10% to 40%
Condom usage rate - high risk	5% Est	0% to 10%
Condom effectiveness	90% [26]	85.0% to 95.0%
**Annual costs (US$)**		
Non-HIV medical costs	200	100 to 300
HIV costs	1,000 [27]	800 to 1200
ART cost	150 [28,29]	100 to 200
PrEP cost	80 [28,29]	50 to 250

ART, antiretroviral therapy; PrEP, pre-exposure prophylaxis.

### Intervention scale-up strategies

We considered strategies where ART, PrEP, or both are scaled up to recruit 25%, 50%, 75% or 100% of remaining eligible individuals annually. For ART scale-up, we considered strategies where ART was offered either to individuals with CD4 cell counts ≤350 cells/μL only (Guidelines), or to all identified HIV-infected individuals (Universal) following a national testing campaign [30]. Thus, the 100% Guidelines strategy models outcomes under full implementation of the current HIV treatment guidelines, whereas the 100% Universal strategy reflects the outcomes under an ideal ‘Universal Test and Treat’ program [12,31].

To investigate the role of PrEP in controlling South Africa’s HIV epidemic, we considered its use in the general adult population (General). We also investigated the health and economic outcomes of using PrEP only in individuals at high-risk of acquiring HIV, defined here as having four or more sexual partners per year and low condom use (Focused).

### Antiretroviral therapy

For the Guidelines strategy, we used a treatment eligibility threshold of 350 cells/μL. In the base case (status quo), 40% of treatment-eligible individuals receive ART, and PrEP is not used [24]. We assumed that 10% of untreated treatment-eligible individuals start ART each year. For the Universal strategy, any infected individual identified and linked to care started ART.

ART is associated with both personal and transmission benefits. Treated individuals have a longer remaining life expectancy than untreated individuals (41.3 remaining years for a 15-year-old if treatment is initiated early, versus 12.2 if untreated) [32,33]. HIV-related mortality rates for each disease stage and ART status are in Additional file 1 – Model details: Table S2. In addition, treated individuals are 95% less likely to infect their sexual partners [9]. We assumed a 2% annual rate of loss-to-follow-up throughout the time horizon. Although studies report a higher rate of loss-to-follow-up in the short term, our estimate is consistent with long-term experience, and the higher efforts to retain patients in care [34,35]. Individuals who discontinue ART do not re-start treatment.

### Pre-exposure prophylaxis

For the General strategies, we assumed that all uninfected adults in the population are eligible to start PrEP. While achieving high rates of PrEP coverage among the entire population may be unfeasible for practical reasons, we explored these possibilities to gain insight and establish an upper bound for the benefits that could be achieved with a large-scale PrEP program. In the Focused strategies, we assumed PrEP is offered only to a population of 3 million uninfected individuals at highest risk for acquiring HIV. This limited approach may render PrEP more feasible. We adjusted the risk profile of the ineligible uninfected population in the Focused strategy to match the overall population risk in the General strategy.

We assumed uninfected individuals taking PrEP have a 60% lower chance of acquiring HIV from an infected sexual partner [15,16]. Because of the uncertainty over PrEP’s real-world effectiveness highlighted in recent trials, we broadly varied PrEP’s effectiveness in the sensitivity analysis (10% to 90%) [17,18,36]. We assumed a 5% annual drop-out rate beyond the non-adherence that is reflected in our estimate of PrEP effectiveness. We also assumed that individuals who were infected while on PrEP and later placed on ART had similar benefits from treatment as those who never received PrEP [15].

### Model structure

We divided South Africa’s adult population (15 to 49 years old, 26.3 million people) into 11 groups defined by HIV disease stage (uninfected, CD4 count >350 cells/μL, 200 to 350 cells/μL, and <200 cells/μL), ART status (untreated, treated, and lost-to-follow-up), and PrEP status (receiving or not receiving PrEP). A schematic diagram of the model is included in Additional file 1 – Model details.

### HIV transmission and progression

We modeled heterosexual HIV transmission based on the average number of annual partnerships per individual, assuming random homogeneous mixing in the population. The annual probability of HIV transmission in a partnership depends on the disease stage and ART use of the infected partner, PrEP use by the uninfected partner, and the rate of condom use and effectiveness.

HIV progression rates prior to and while receiving ART were based on published studies from South Africa [32,33]. ART was assumed to reduce mortality and lower infectivity. We assumed no significant differences in disease progression rates for individuals who previously received PrEP or for those who discontinued ART versus individuals who were never treated.

### Model calibration

Our model was calibrated to match information about South Africa’s HIV epidemic, including initial HIV prevalence (17.9%), HIV incidence (1.4%), and population growth [24,37]. Prevalence in individuals entering the model at age 15 was estimated at 5% [25]. Using this data, we estimated that HIV prevalence will decline to 10.4% over the next 20 years, due to a reduction in incidence from 1.4% to 0.8%, while population growth will average 1.1% annually.

### Health outcomes and costs

We measured costs and QALYs for each strategy over 20 years, discounted at 3% annually. We included costs and benefits incurred over the time horizon, plus discounted future lifetime costs and QALYs incurred by the population at the end of the time horizon.

We assumed lower quality of life as HIV progresses, and that ART increases quality of life by 10% of the difference between uninfected and infected untreated individuals, to include both the benefits and the side effects of ART.

We assumed annual ART costs of $150, and annual PrEP costs of $80, reflecting the reduced prices of HIV drugs accessible in South Africa [28,29]. All individuals incurred an annual non-HIV medical cost of $200. In addition, HIV-infected individuals incurred $1,000 in HIV-related annual medical costs [27].

## Results

Our model suggests that, under the status quo, 5,400,000 individuals will become newly infected, 5,600,000 infected individuals will die of HIV-related causes, and 1,900,000 infected individuals will start ART over the next 20 years. Table 2 summarizes the results of the key scale-up strategies considered.

**Table 2:** Outcomes over 20 years of various strategies to scale up single or combination HIV prevention and treatment programs: Guidelines antiretroviral therapy (individuals with CD4 cell counts ≤350 cells/μL only), Universal antiretroviral therapy (all HIV-infected individuals), General pre-exposure prophylaxis (general population), Focused pre-exposure prophylaxis (individuals at high risk of acquiring HIV)

**ART Guidelines portfolios**	**Base case**	**(50% Guidelines ART, 0% General PrEP)**	**(50% Guidelines ART, 50% General PrEP)**	**(50% Guidelines ART, 100% General PrEP)**	**(100% Guidelines ART, 0% General PrEP)**	**(100% Guidelines ART, 50% General PrEP)**	**(100% Guidelines ART, 100% General PrEP)**
**Total Population**	28,899,757	30,584,812	31,140,505	31,243,269	31,155,266	31,559,954	31,637,147
**HIV population**	3,010,186	2,952,938	1,957,738	1,857,875	2,924,495	2,038,587	1,946,176
**HIV prevalence**	10.4%	9.7%	6.3%	5.9%	9.4%	6.5%	6.2%
**PrEP entry**	0%	0%	50%	100%	0%	50%	100%
**ART entry**							
**(late and advanced)**	10%	50%	50%	50%	100%	100%	100%
**ART entry**							
**(early)**	0%	0%	0%	0%	0%	0%	0%
**Started ART**	1,898,565	4,291,707	3,525,062	3,400,388	4,983,325	4,178,534	4,048,308
**Total QALYs**							
**(millions)**	939	982	1,003	1,007	998	1,015	1,017
**Total costs**							
**($ billions)**	$282.2	$299.8	$343.5	$349.0	$306.0	$350.8	$356.5
**Infections averted**		1,512,972	3,545,397	3,826,716	2,030,990	3,751,962	3,993,700
**CE ratio**		$413	$958	$992	$408	$914	$954
**ART Universal portfolios**	**Base case**	**(50% Universal ART, 0% General PrEP)**	**(50% Universal ART, 50% General PrEP)**	**(50% Universal, 100% General PrEP)**	**(100% Universal ART, 0% General PrEP)**	**(100% Universal ART, 50% General PrEP)**	**(100% Universal ART, 100% General PrEP)**
**Total Population**	28,899,757	31,957,916	32,099,883	32,135,197	32,344,976	32,424,302	32,444,183
**HIV population**	3,010,186	2,429,248	2,020,691	1,965,384	2,372,222	2,045,369	2,002,584
**HIV prevalence**	10.4%	7.6%	6.3%	6.1%	7.3%	6.3%	6.2%
**PrEP entry**	0%	0%	50%	100%	0%	50%	100%
**ART entry**							
**(late and advanced)**	10%	50%	50%	50%	100%	100%	100%
**ART entry**							
**(early)**	0%	50%	50%	50%	100%	100%	100%
**Started ART**	1,898,565	5,605,789	4,997,687	4,883,560	5,946,284	5,442,264	5,354,623
**Total QALYs**							
**(millions)**	939	1,025	1,031	1,033	1,037	1,041	1,042
**Total costs**							
**($ billions)**	$282.2	$309.1	$360.2	$366.2	$312.3	$365.7	$372.1
**Infections averted**		3,591,057	4,356,210	4,494,263	4,014,941	4,579,560	4,675,735
**CE ratio**		$314	$849	$902	$307	$819	$876
**Focused PrEP portfolios**	**Base case**	**(10% Guidelines ART, 50% Focused PrEP)**	**(10% Guidelines ART, 100% Focused PrEP)**	**(50% Guidelines ART, 100% Focused PrEP)**	**(100% Guidelines ART, 100% Focused PrEP)**	**(50% Universal ART, 100% Focused PrEP)**	**(100% Universal ART, 100% Focused PrEP)**
**Total Population**	28,899,757	29,582,098	30,075,564	31,218,742	31,624,528	32,147,612	32,457,488
**HIV population**	3,010,186	2,232,122	1,743,582	1,983,526	2,059,891	2,010,021	2,038,731
**HIV prevalence**	10.4%	7.55%	5.8%	6.4%	6.5%	6.3%	6.3%
**PrEP entry**	0%	50%	100%	100%	100%	100%	100%
**ART entry**							
**(late and advanced)**	10%	10%	10%	50%	100%	50%	100%
**ART entry**							
**(early)**	0%	0%	0%	0%	0%	50%	100%
**Started ART**	1,898,565	1,639,299	1,454,123	3,455,184	4,104,640	4,897,716	5,362,426
**Total QALYs**							
**(millions)**	939	962	978	1,006	1,016	1,033	1,042
**Total costs**							
**($ billions)**	$282.2	$277.1	274.9	292.9	299.8	307.9	313.3
**Infections averted**		1,837,744	3,084,508	3,642,543	3,840,111	4,468,827	4,663,411
**CE ratio**		Cost saving	Cost saving	$163	$229	$276	$302
**General PrEP portfolios**	**Base case**	**(10% Guidelines ART, 50% General PrEP)**	**(10% Guidelines ART, 100% General PrEP)**				
**Total Population**	28,899,757	29,970,799	30,151,535				
**HIV population**	3,010,186	1,693,209	1,579,445				
**HIV prevalence**	10.4%	5.65%	5.24%				
**PrEP entry**	0%	50%	100%				
**ART entry**							
**(late and advanced)**	10%	10%	10%				
**ART entry**							
**(early)**	0%	0%	0%				
**Started ART**	1,898,565	1,485,240	1,420,126				
**Total QALYs**							
**(millions)**	939	975	980				
**Total costs**							
**($ billions)**	$282.2	$323.9	$329.6				
**Infections averted**		2,998,344	3,381,214				
**CE ratio**		$1,172	$1,158				

ART, antiretroviral therapy; CE, cost -effectiveness; PrEP, pre-exposure prophylaxis; QALY, quality-adjusted life-year.

### Health benefits of scaling up ART and PrEP

**Figure 1:**
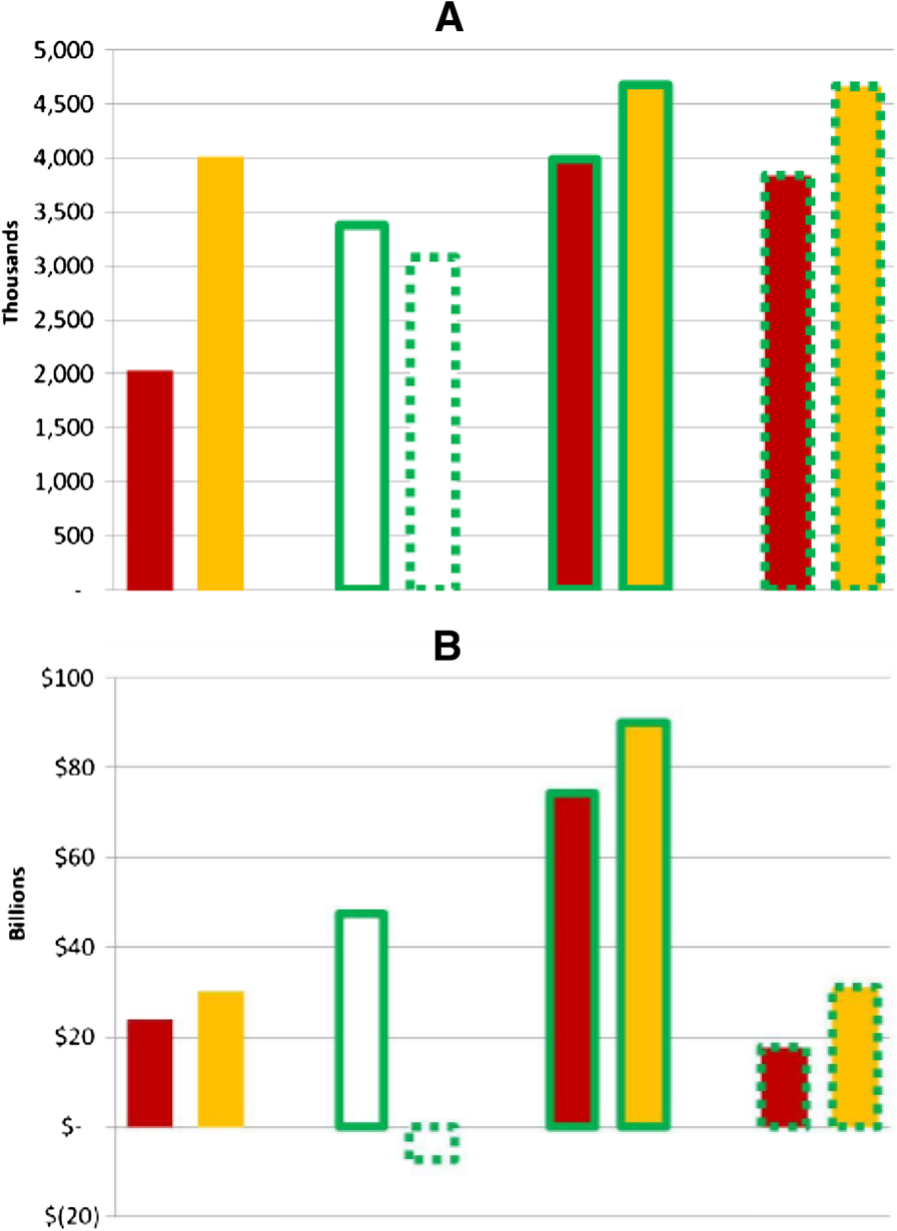
**HIV infections averted and incremental costs versus the status quo with 100% scale-up for single or combination programs over 20 years. (A)** Infections averted over 20 years with 100% scale-up for single or combination programs. **(B)** Incremental costs versus status quo over 20 years with 100% scale-up for single or combination programs. Strategies include: Guidelines ART (individuals with CD4 cell counts ≤350 cells/μL only), Universal ART (all HIV-infected individuals), General PrEP (general population), and Focused PrEP (individuals at high risk of acquiring HIV). Bars 1 and 2 show results of ART programs alone, 3 and 4 show results of PrEP programs alone, and bars 5 to 8 show results of pairwise combinations of ART and PrEP programs. ART strategies indicated by bar color: Guidelines - red; Universal - orange; Status quo - no color. PrEP strategies indicated by bar outline: General - solid line; Focused - dashed line; Status quo - no line.

Figure 1 presents the number of HIV infections averted and incremental costs versus the status quo for 100% scale-up of all strategies considered, individually and in combination. In general, strategies involving Universal ART scale-up avert more infections than strategies involving General PrEP for the same rate of scale-up.

In the extreme scenario of 100% Universal ART without PrEP, 75% of new infections are averted over 20 years, whereas 100% coverage with Guidelines ART averts 38% of new infections. Without PrEP, up to 50% more people start treatment in the Universal ART strategies compared to the corresponding Guidelines strategies, and 2 to 3.4 times as many infections are averted, depending on program scale (Table 2).

**Figure 2:**
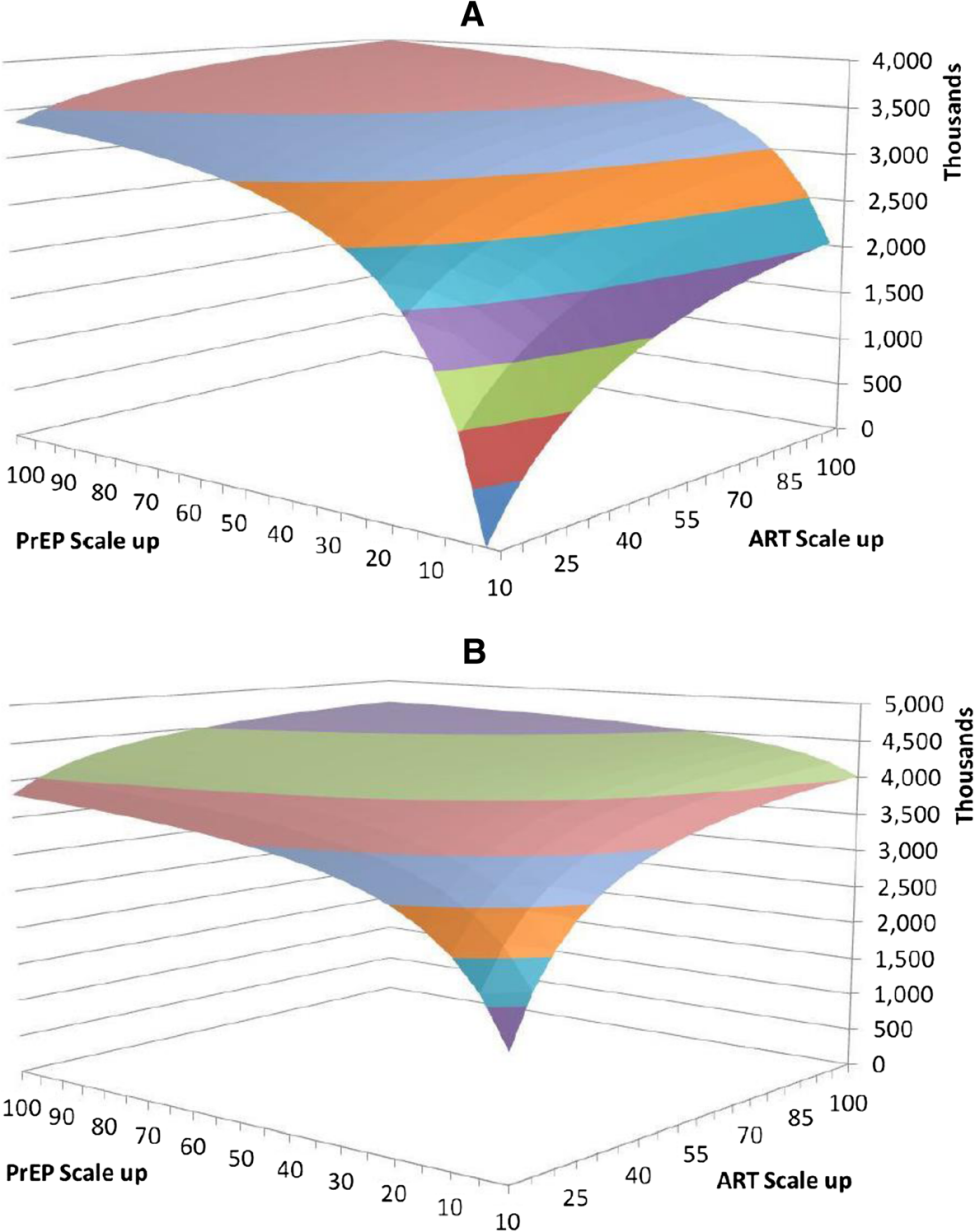
**Infections averted over 20 years with portfolios of scaled-up ART and PrEP. (A)** Infections averted over 20 years with portfolios of scaled-up Guidelines ART (individuals with CD4 cell counts ≤ 350 cells/μL only) and General PrEP (general population). **(B)** Infections averted over 20 years with portfolios of scaled up Universal ART (all HIV-infected individuals) and General PrEP (general population).

Scaling up General PrEP alone to 100% while keeping ART at current recruitment levels would avert 63% of new infections over 20 years. Most of PrEP’s benefits are achieved with General PrEP programs with less aggressive recruitment rates; increasing PrEP recruitment rate from 50% to 100% in the general population for any given ART level averts only an additional 2% to 7% of new infections. This is because a significant proportion of uninfected individuals will start PrEP over the time horizon even with a less aggressive program recruitment, and the incremental benefits of an initially less aggressive program approach the benefits of a more aggressive program over time. Figure 2 shows the infections averted by all combinations of General PrEP and Guidelines ART (Figure 2A) or Universal ART (Figure 2B). Note that as indicated in Figure 2B, a 10% Universal ART program offers incremental benefits versus the status quo due to expanded ART eligibility criteria (1,530,000 infections averted); adding General PrEP averts up to an additional 2,270,000 infections.

Implementing PrEP in 50% of the uninfected population in the presence of Guidelines ART programs averted 61% to 69% of new infections, depending on the scale of the ART program, versus 16% to 38% for Guidelines ART alone. The marginal benefit of PrEP was smaller when added to Universal ART programs, since treating those with early HIV reduces the size of the population that transmits HIV. Thus, 50% PrEP incremental to Universal ART strategies averted 75% to 85% of new infections, versus 53% to 75% for Universal ART alone. This suggests that Universal ART and General PrEP function as partial substitutes and the benefits of PrEP may be more significant if Universal ART cannot be accomplished. If it is achievable, Focused PrEP can be an effective intervention: 100% Focused PrEP required reaching 10% of the adult population, and averted 57% of all new infections even without any further ART scale-up. When added to 50% ART programs, Focused PrEP averted a significant number of additional infections. Scaling up to 50% Guidelines ART and 100% Focused PrEP averted 2.4 times as many infections as the ART program alone. These effects were smaller for larger ART programs and Universal ART, but in all scenarios Focused PrEP averted 20% or more incremental HIV infections compared to the ART program alone (Table 2). These results, however, critically depend on the ability to identify the high-risk population and the effectiveness of PrEP in this group, both of which are uncertain.

### Cost-effectiveness

**Figure 3:**
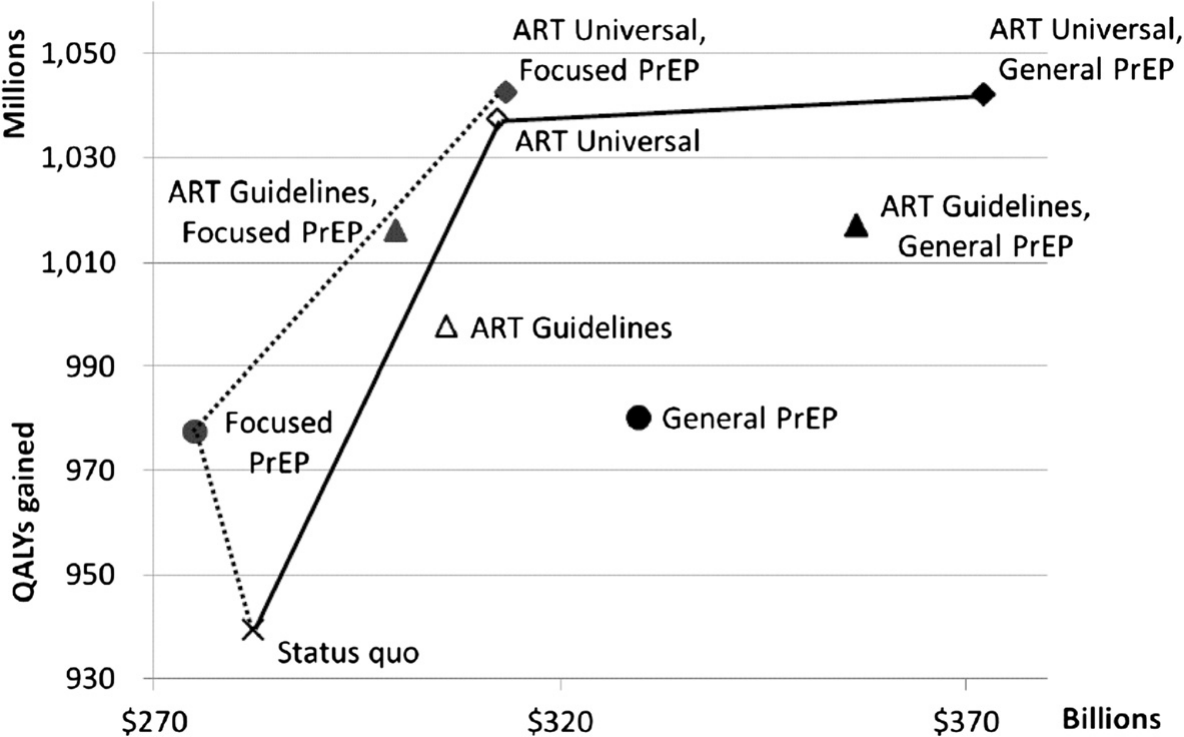
**Cost-effectiveness (quality-adjusted life-years versus total costs over 20 years) of 100% scale-up for single or combination programs.** Guidelines ART (individuals with CD4 cell counts ≤350 cells/μL only), Universal ART (all HIV-infected individuals), General PrEP (general population), Focused PrEP (individuals at high risk of acquiring HIV). ART strategies indicated by dot shape: Guidelines - triangle; Universal - diamond; Status quo - X mark. PrEP strategies indicated by dot color: General - black; Focused - gray. There is no PrEP in the status quo.

Universal ART had higher total costs, but also added significantly more QALYs than Guidelines ART: Universal ART cost $310 to $320 per QALY gained versus the status quo, whereas Guidelines ART scale-up cost $410 to $420 per QALY gained versus the status quo, with total costs as well as benefits lower than the corresponding Universal ART strategy. Figure 3 shows the benefits (QALYs) and costs for single and combination programs (Guidelines and Universal ART, General and Focused PrEP).

With our base case assumptions of PrEP effectiveness, strategies consisting of Focused PrEP alone were cost-saving compared to the status quo and offered significant benefits. Combinations of Focused PrEP and ART dominated ART alone, offering greater benefits at lower costs: Universal ART scale-up to 100% in addition to Focused PrEP cost $150 per QALY gained. This was also the most effective of all strategies (Table 2). However, if Focused PrEP is infeasible, Universal ART is the alternative with the most attractive cost-effectiveness profile.

We estimated that General PrEP strategies would cost $1,200 per QALY gained compared to the status quo. For similar levels of benefit, General PrEP strategies were more costly than ART programs. Adding 50% General PrEP to Guidelines ART scenarios gained QALYs versus ART alone at costs between $1,600 per QALY gained (for 25% Guidelines ART) and $2,650 per QALY gained ( for 100% Guidelines ART). When added to Universal ART, 50% General PrEP added QALYs at $4,300 per QALY gained (25% Universal), but becomes more expensive at $13,300 per QALY gained for larger ART programs (100% Universal).

While all strategies met traditional thresholds of cost-effectiveness, our analysis suggests that some combinations of strategies are much more attractive, given South Africa’s gross domestic product per capita of $10,700 [38].

### Sensitivity analysis

We performed extensive one-way sensitivity analyses on all model parameters. We found that the results varied with the effectiveness of the two interventions, discontinuation rate for both, the cost of PrEP, the number of sexual partners, and the rates of condom usage in the population. However, the same alternatives were preferred in terms of infections averted and cost-effectiveness. In particular, we found that a PrEP effectiveness of 10% limited the cost savings of Focused PrEP by 60% and reduced the cost-effectiveness of General PrEP by 50%.

Higher rates of PrEP attrition played little role in the effects of the programs, assuming enough recruitment to the program occurred to compensate for the attrition. Higher rates of ART attrition (which made individuals ineligible for ART) did not significantly decrease the cost-effectiveness of ART scale-up, even at relatively high levels (10% versus 2% in the base case).

The Focused PrEP strategy was no longer cost-saving for costs of PrEP above $150. This price increase may reflect additional costs to target high-risk individuals, improve adherence, and monitor for adverse effects of PrEP. To be as cost-effective as universal ART ($310 per QALY), Focused PrEP annual cost would be $260 per person, and $580 to cost as much per QALY ($1,200 per QALY) as General PrEP at $80 in the base case.

## Discussion and conclusions

We have presented an analysis of the effectiveness and cost-effectiveness of two promising antiretroviral-based HIV prevention technologies: ART and PrEP. We found that targeting PrEP to individuals at particularly high risk of infection may be cost-saving, but that if identifying high-risk HIV-negative individuals is not practical, then implementing universal treatment, regardless of scale, would be the most cost-effective ART intervention to reduce HIV transmission in South Africa. Our analysis is unique in considering the joint outcomes and cost-effectiveness of implementing ART and PrEP for HIV control.

We found that scaling up ART programs provided greater value than untargeted PrEP programs. Compared with the status quo, scaling up ART, either according to current guidelines or with universal treatment, appeared more cost-effective than scaling up untargeted PrEP. Moreover, implementing PrEP in the general population was never preferable to increasing ART coverage before ART coverage reached 100%.

We have also shown that scaling up ART according to current guidelines is less cost-effective than scaling up universal treatment. Universal treatment averted more infections than current guidelines even at relatively small program scales. Scaling up ART according to guidelines is still considered cost-effective in South Africa according to thresholds articulated in the Commission on Macroeconomics and Health [38]. However, universal treatment was associated with a greater number of infections averted and a greater gain in QALYs for each unit investment in resources relative to the status quo, and the results got increasingly attractive as we considered longer planning horizons. When looking at the results for 10 years, Universal ART appeared less favorable (while still cost-effective), at $490 per QALY gained versus status quo. Taking into account benefits and costs over 20 years, this cost was $310 per QALY. Hence, Universal ART becomes more attractive if we consider the long-term impact, and the effects may become even stronger with longer horizons. We chose to use a 20-year time horizon because, unlike longer horizons, this time frame is still considered relevant for practical decision-making.

Our analysis suggests that despite the effectiveness of oral PrEP, its costs make it a relatively low-value alternative if used in the general population. If, however, it can be targeted to individuals at higher risk of acquiring HIV and adherence can be maintained, then PrEP can potentially be cost-saving. The effectiveness of PrEP remains uncertain, as suggested by the recent mixed results of clinical trials and the challenges with adherence. Our sensitivity analyses showed that providing PrEP to high-risk populations can be cost-saving as long as PrEP effectiveness is greater than 10%.

Our analysis has clear implications for resource prioritization between ART and PrEP; however, its limitations are important to note. Scaling up ART or PrEP programs requires a substantial budget investment at a time of global economic uncertainty. Our analysis cannot determine whether these resources would be available, only that some investments provide greater returns than others (for example, Universal ART relative to Guidelines ART). For those reasons, we explored a wide range of implementation scales. In addition, there is considerable uncertainty associated with the long-term epidemic impact of universal treatment [39]. To explore this issue, we performed sensitivity analyses on parameters affecting the effectiveness of ART programs, such as reduction in infectivity if receiving ART, and the rates of attrition from the program. Additional uncertainty is related to the potential consequences of sustained PrEP, both in terms of individual toxicities and its implications for resistance and future treatment options. As more data become available, model assumptions and structure may need to be further refined to incorporate these findings. Finally, our model was based on South African data, where more people live with HIV than any other country. Our estimates of the relative effectiveness of alternative approaches can be generalized to many other areas of southern Africa, but additional work would be needed to apply this work to regions or countries with a different epidemic pattern and different costs of health care.

A proliferation of new strategies to control HIV are developed and tested in South Africa, but economic circumstances require judicious use of scarce resources. Developed countries have recently updated guidelines to recommend universal ART, and the Center for Disease Control has provided guidance on the use of PrEP in men who have sex with men [40,41]. Our analysis supports the new World Health Organization recommendations on ART for sero-discordant couples, and estimates the value of extending universal treatment to all infected individuals [42]. Our work provides new insights into the joint effects of ART and PrEP in a generalized HIV epidemic, and the cost-effectiveness of strategies for program scale-up in developing countries. Implementing the strategies outlined by these models can lead to better use of scarce resources and can prevent a significant proportion of new HIV infections, in the long term leading to epidemic control.

## Competing interests

The authors declare that they have no competing interests.

## Authors’ contributions

SSA identified parameters, designed and implemented the model, analyzed the results and wrote the manuscript. PMG provided critical guidance on intervention effectiveness, helped with the model design, and edited the manuscript. EB provided parameters, designed the model, analyzed the results and edited the manuscript. All authors have reviewed and approved this version of the manuscript.

## Supplementary Material

Additional file 1 Model details. Click here for file
