# Supplementary material for: Comparative effectiveness and cost-effectiveness of antiretroviral therapy and pre-exposure prophylaxis for HIV prevention in South Africa
Source: BMC Med. 2014 Mar 17;12:46. doi: 10.1186/1741-7015-12-46 (PMC4003813; doi:10.1186/1741-7015-12-46)
Supplement: Additional file 1 — Model details. [file 1741-7015-12-46-S1.doc]

**Additional file 1**

**Introduction**

We show a diagram of the dynamic compartmental model in Additional file 1 Figure 1. We denote by *N_i_*(*t*) the number of individuals in compartment *N* at time *t*. *i* represents the disease status. Full model notation is summarized in Additional file 1 Table 1.

Additional file 1 Table 2 lists the values, ranges, and sources for parameters. Individuals enter/exit the population and transition between compartments with rates that depend on demographic data, disease progression parameters, and program scale (ART, PrEP). All rates are evaluated yearly.

**Population dynamics**

Individuals enter the model at age 15 (14 year olds turning 15), either uninfected at rate *e*(*U_u_*),or already infected at rate *e*(*U_e_*), since HIV prevalence in 15 year olds is 5% . Hence, the total rate of entry into the population is *e*(*U_u_*)+*e*(*U_e_*). Exit from the model occurs when individuals in all compartments die of non-HIV causes (rates denoted as *d*(*N_i_*)), or of HIV-related causes (rates denoted as *a*(*N_i_*)) from all compartments except *U_u_* and *P_u_*. Maturation occurs from all compartments at age 49 with rate *m*(*N_i_*).

**HIV progression**

Disease progression occurs with rates *p*(*N_i_*) that depend on disease stage *i* and ART status as indicated by *N*. These parameters are obtained from published models of the natural history of HIV by taking reciprocals of typical time spent in each disease stage. These times are higher (hence the rates *p*(*N_i_*) are lower) if individuals are on ART. ART also reduces the AIDS death rate *a*(*N_i_*). Individuals with more advanced disease are more infectious.

**HIV transmission**

HIV acquisition by uninfected individuals occurs via risky sexual contacts with infected individuals. We assume that condom use is 90% effective (*Eff*=*90%*)in reducing sexual contact risk, and that condoms are used in 25% of contacts (*u*(*N_i_*)=*25%*) . The number new infections at time *t* is determined based on the sufficient contact rates for sexual disease transmission *c*(*N_i_*,*M_j_*), which are defined for the uninfected compartments *P_u_* and *U_u_*. Sexual contacts are considered no risk if a condom was effectively used, low risk if a condom was used but was ineffective and high risk if a condom was not used. The contact rates are a product of the number of yearly sexual partners *k*(*N_i_*), the probability of using a condom and failing *u*(*N_i_*)x(*1*-*Eff*) for low risk or the probability of not using a condom at all *1*-*u*(*N_i_*) for high risk, the probability of HIV acquisition per partnership *s(N_i_,M_j_)*, and the probability *f(M_j_)* of choosing a sexual partner from compartment *M_j_*.

$$c(N_{i},M_{j})=c^{L}(N_{i},M_{j})+c^{H}(N_{i},M_{j})$$

$$c^{L}(N_{i,}M_{j})=k(N_{i})\times u(N_{i})\times(1-Eff)\times s(N_{i},M_{j})\times f(M_{j})$$

$$c^{H}(N_{i,}M_{j})=k(N_{i})\times(1-u(N_{i}))\times s(N_{i},M_{j})\times f(M_{j})$$

$$f(M_{j})=\frac{k(M_{j})\times M_{j}(t)}{\sum_{\forall N,h} k(N_{h})\times N_{h}(t)}; M\in\left\{P,U,T,Q\right\}$$

**ART program**

In scaling up ART according to the Guidelines, only individuals with late and advanced HIV (CD4 T-cell counts below 350 cells/µL) can be recruited into the program, thus transitioning from compartments *U_l_* and *U_a_* to the corresponding treated compartments *T_l_* and *T_a_*. For Universal ART strategies, individuals with early HIV (CD4 T-cell counts above 350 cells/µL) are also eligible for ART and can transition from compartment *U_e_* to *T_e_*.

According to program scale and eligibility criteria, at time *t* a fraction *h*(*U_i_*) of individuals in compartment *U_i_*(*t*) start ART. Treated individuals can also quit ART at a rate *q*(*T_i_*) and move to the corresponding compartment *Q_i_*, in which case they are no longer eligible for recruitment into ART.

ART lowers HIV progression rates *p*(*T_i_*), HIV-related death rates *a*(*T_i_*) and the infectivity of treated individuals by 95% .

**PrEP program**

We considered two strategies for using PrEP: General and Focused. All individuals receiving PrEP are 60% less likely to acquire HIV in the case of a risky contact . For the General strategy, all uninfected individuals are eligible for recruitment into PrEP. Uninfected individuals in compartment *U_u_* transition to compartment *P_u_* at rates *r*(*U_u_*) determined by program scale.

For the Focused strategy, we assume only a population of high risk individuals representing 10% of the total population are recruited into PrEP. High risk individuals are defined as having a higher yearly number of partnerships (4 versus 1.2 for the general population) and lower rates of condom use (5% versus 25% in the general population). We assume the program can correctly identify and recruit these individuals into PrEP so that the number of individuals receiving PrEP is approximately constant.

For both strategies, individuals leave the PrEP program at rate *q*(*P_u_*)=*5%* yearly.

**Model equations**

To project the epidemic forward in time, we use a system of non-linear differential equations as described below. The total population in the model at time *t* is:

$$X(t)=\sum_{N\in\left\{P,U,T,Q\right\}} \sum_{i\in\left\{u,e,l,a\right\}} N_{i}(t)$$

Equation governing the change in time for individuals on PrEP:

$$\frac{dP_{u}(t)}{dt}=-P_{u}(t)\left[m(P_{u})+d(P_{u})+\sum_{M\in\left\{U,T,Q\right\}} \sum_{j\in\left\{e,l,a\right\}} c(P_{u},M_{j})(t)\right]+U_{u}(t)\times r(U_{u})$$

Equation governing the change in time for uninfected individuals:

$$\begin{matrix} \frac{dU_{u}(t)}{dt}=e(U_{u})\times X(t)\times(1-prev_{15})-U_{u}(t)\left[m(U_{u})+d(U_{u})+r(U_{u})+\sum_{M\in\left\{U,T,Q\right\}} \sum_{j\in\left\{e,l,a\right\}} c(U_{u},M_{j})(t)\right] \\ +P_{u}(t)\times q(P_{u}) \end{matrix}$$

Equation governing the change in time for untreated individuals with early HIV:

$$\begin{matrix} \frac{dU_{e}(t)}{dt}=e(U_{u})\times X(t)\times prev_{15}+U_{u}(t)\left[\sum_{M\in\left\{U,T,Q\right\}} \sum_{j\in\left\{e,l,a\right\}} c(U_{u},M_{j})(t)\right]+P_{u}(t)\left[\sum_{M\in\left\{U,T,Q\right\}} \sum_{j\in\left\{e,l,a\right\}} c(P_{u},M_{j})(t)\right] \\ -U_{e}(t)\left[m(U_{e})+d(U_{e})+a(U_{e})+p(U_{e})+h(U_{e})\right] \end{matrix}$$

Equation governing the change in time for untreated individuals with late HIV:

$$\frac{dU_{l}(t)}{dt}=U_{e}(t)\times p(U_{e})-U_{l}(t)\left[m(U_{l})+d(U_{l})+a(U_{l})+p(U_{l})+h(U_{l})\right]$$

Equation governing the change in time for untreated individuals with advanced HIV:

$$\frac{dU_{a}(t)}{dt}=U_{l}(t)\times p(U_{l})-U_{a}(t)\left[m(U_{a})+d(U_{a})+a(U_{a})+h(U_{a})\right]$$

Equation governing the change in time for treated individuals with early HIV:

$$\frac{dT_{e}(t)}{dt}=U_{e}(t)\times h(U_{e})-T_{e}(t)\left[m(T_{e})+d(T_{e})+a(T_{e})+p(T_{e})+q(T_{e})\right]$$

Equation governing the change in time for treated individuals with late HIV:

$$\frac{dT_{l}(t)}{dt}=T_{e}(t)\times p(T_{e})+U_{l}(t)\times h(U_{l})-T_{l}(t)\left[m(T_{l})+d(T_{l})+a(T_{l})+p(T_{l})+q(T_{l})\right]$$

Equation governing the change in time for treated individuals with advanced HIV:

$$\frac{dT_{a}(t)}{dt}=T_{l}(t)\times p(T_{l})+U_{a}(t)\times h(U_{a})-T_{a}(t)\left[m(T_{a})+d(T_{a})+a(T_{a})+q(T_{a})\right]$$

Equation governing the change in time for individuals with early HIV who quit ART:

$$\frac{dQ_{e}(t)}{dt}=T_{e}(t)\times q(T_{e})-Q_{e}(t)\left[m(Q_{e})+d(Q_{e})+a(Q_{e})+p(Q_{e})\right]$$

Equation governing the change in time for individuals with late HIV who quit ART:

$$\frac{dQ_{l}(t)}{dt}=Q_{e}(t)\times p(Q_{e})+T_{l}(t)\times q(T_{l})-Q_{l}(t)\left[m(Q_{l})+d(Q_{l})+a(Q_{l})+p(Q_{l})\right]$$

Equation governing the change in time for individuals with advanced HIV who quit ART:

$$\frac{dQ_{a}(t)}{dt}=Q_{l}(t)\times p(Q_{l})+T_{a}(t)\times q(T_{a})-Q_{a}(t)\left[m(Q_{a})+d(Q_{a})+a(Q_{a})\right]$$

**Health outcomes and costs**

Additional file 1 Table 3 summarizes the results of key strategies for program scale up considered.

The infections averted in each strategy were calculated by subtracting the number of new infections occurring over the time horizon for the considered strategy from the number of new infections occurring in the status quo.

To evaluate cost-effectiveness, we measured QALYs and costs in US dollars over 20 years, discounted at 3% annual interest rate for all strategies. The incremental cost-effectiveness ratio (ICER) for any strategy can be obtained as the ratio of incremental costs to incremental QALYs gained:

*ICER*_strategy_ =$\left[\frac{TotalCost_{\text{ Strategy}}-TotalCost_{\text{Status quo}}}{QALYs_{\text{Strategy}}-QALYs_{\text{Status quo}}}\right]$

**HIV prevalence**

HIV prevalence was computed according to the formula:

*HIV Prevalence* *=* $\left[\frac{\sum_{N\in\left\{U,T,Q\right\}} \sum_{i\in\left\{e,l,a\right\}} N_{i}(t)}{\sum_{N\in\left\{P,U,T,Q\right\}} \sum_{i\in\left\{u,e,l,a\right\}} N_{i}(t)}\right]$

In the status quo, HIV prevalence is predicted to decline from 17.9% to 10.4% due to high AIDS mortality, the natural course of the disease and ART scale up efforts. 100% PrEP strategies further reduced prevalence to 5.2% (General) and 5.8% (Focused). Because ART keeps infected people alive, the final prevalence was larger for ART strategies: 9.4% (Guidelines) and 7.3% (Universal). Adding PrEP to ART provided additional reductions in prevalence, and the effect was smaller for larger ART programs and for Universal ART.

**Additional file 1 Table 1 – Model compartments and** notation

| **Compartment** | **u (***N_i_=P_u_*) |  |  |  |
| --- | --- | --- | --- | --- |
| **P** | Uninfected |  |  |  |
|  | PrEP |  |  |  |
| **Compartment** | **u (***N_i_=U_u_*) | **e (***N_i_=U_e_*) | **l (***N_i_=U_l_*) | **a (***N_i_=U_a_*) |
| **U** | Uninfected | Early HIV | Late HIV | Advanced HIV |
|  | No ART | No ART | No ART | No ART |
| **Compartment** |  | **e (***N_i_=T_e_*) | **l (***N_i_=T_l_*) | **a (***N_i_=T_a_*) |
| **T** |  | Early HIV | Late HIV | Advanced HIV |
|  |  | ART | ART | ART |
| **Compartment** |  | **e (***N_i_=Q_e_*) | **l (***N_i_=Q_l_*) | **a (***N_i_=Q_a_*) |
| **Q** |  | Early HIV | Late HIV | Advanced HIV |
|  |  | Quit ART | Quit ART | Quit ART |

**Additional file 1** **Table 2**: Detailed list of model parameters with source and ranges (disease, epidemic and behavioral characteristics, intervention scale and effectiveness, health and intervention costs)

| **Parameter** | **Value and source** | **Range** |
| --- | --- | --- |
| **HIV Prevalence** |  |  |
| Overall HIV prevalence | 17.9% | 17.2%-18.3% |
| HIV prevalence in 15 year olds | 5.0% | 3%-7% |
| **Initial disease stage** |  |  |
| Early HIV | 50% | 45%-55% |
| Late HIV | 28% | 23%-33% |
| Advanced HIV | 22% | 17%-27% |
| **Population dynamics** |  |  |
| Entry rate | 0.057 ,Calc | 0.050-0.064 |
| Maturation rate | 0.032 ,Calc | 0.028-0.036 |
| Non-HIV related death rate | 0.01 ,Calc | 0.008-0.012 |
| **HIV progression rates (annual)** |  |  |
| Early HIV to late HIV- no ART | 0.164 | 0.150-0.178 |
| Late HIV to advanced HIV- no ART | 0.260 | 0.230-0.290 |
| Early HIV to late HIV- ART | 0.062 | 0.050-0.074 |
| Late HIV to advanced HIV- ART | 0.062 | 0.050-0.074 |
| **HIV-related death rates** |  |  |
| Early HIV- no ART | 0.008 | 0.006-0.010 |
| Late HIV- no ART | 0.090 | 0.080-0.100 |
| Advanced HIV- no ART | 0.450 | 0.400-0.500 |
| Early HIV- ART | 0.001 | 0.0008-0.0012 |
| Late HIV- ART | 0.018 | 0.015-0.021 |
| Advanced HIV- ART | 0.110 | 0.100-0.120 |
| **ART** |  |  |
| Initial access to ART – late HIV | 29% | 25%-33% |
| Initial access to ART – advanced HIV | 54% | 50%-58% |
| ART quit rate (annual) | 0.02 Est | 0-0.20 |
| Sexual transmission reduction | 95% | 50.0%-99.0% |
| **PrEP** |  |  |
| PrEP quit rate (annual) | 0.05 Est | 0-0.20 |
| Sexual acquisition reduction | 60% | 10%-90% |
| **Sexual behavior** |  |  |
| Number of sexual partners per year – general | 1.5 ,Est | 1-2 |
| Number of sexual partners per year – high risk | 4 Est | 3-5 |
| Condom usage rate - general | 25% ,Est | 10%-40% |
| Condom usage rate - high risk | 5% Est | 0%-10% |
| Condom effectiveness | 90% | 85.0%-95.0% |
| **Sexual transmission of HIV per partnership** |  |  |
| Early HIV- no ART | 0.075 | 0.065-0.085 |
| Late HIV- no ART | 0.115 | 0.125-0.105 |
| Advanced HIV- no ART | 0.150 | 0.13-0.17 |
| Early HIV- ART | 0.004 | 0.003-0.005 |
| Late HIV- ART | 0.006 | 0.005-0.007 |
| Advanced HIV- ART | 0.008 | 0.007-0.009 |
| **Quality-of-life adjustments** |  |  |
| Uninfected | 1 | 0.95-1 |
| Early HIV – no ART | 0.9 | 0.85-0.95 |
| Late HIV – no ART | 0.8 | 0.75-0.85 |
| Advanced HIV – no ART | 0.7 | 0.65-0.75 |
| Increase if on ART | 15% | 0%-30% |
| **Annual costs ($)** |  |  |
| Non-HIV medical costs | 200 | 100-300 |
| HIV costs | 1,000 | 800-1200 |
| ART cost | 150 | 100-200 |
| PrEP cost | 80 | 50-250 |

| **ART Guidelines** | **Base case** | **(25 G,**  **0 Gen)** | **(25 G,**  **50 Gen)** | **(25 G,**  **100 Gen)** | **(50 G,**  **0 Gen)** | **(50 G,**  **50 Gen)** | **(50 G,**  **100 Gen)** | **(75 G,**  **0 Gen)** | **(75 G,**  **50 Gen)** | **(75 G,**  **100 Gen)** | **(100 G,**  **0 Gen)** | **(100 G,**  **50 Gen)** | **(100 G, 100 Gen)** |
| --- | --- | --- | --- | --- | --- | --- | --- | --- | --- | --- | --- | --- | --- |
| **Total Population** | 28,899,757 | 29,844,974 | 30,617,260 | 30,753,911 | 30,584,812 | 31,140,505 | 31,243,269 | 30,945,154 | 31,403,450 | 31,489,984 | 31,155,266 | 31,559,954 | 31,637,147 |
| **HIV population** | 3,010,186 | 2,983,731 | 1,845,951 | 1,738,789 | 2,952,938 | 1,957,738 | 1,857,875 | 2,935,331 | 2,009,419 | 1,914,049 | 2,924,495 | 2,038,587 | 1,946,176 |
| **HIV prevalence** | 10.4% | 10.0% | 6.0% | 5.7% | 9.7% | 6.3% | 5.9% | 9.5% | 6.4% | 6.1% | 9.4% | 6.5% | 6.2% |
| **PrEP entry** | 0% | 0% | 50% | 100% | 0% | 50% | 100% | 0% | 50% | 100% | 0% | 50% | 100% |
| **ART entry**  **(late & advanced)** | 10% | 25% | 25% | 25% | 50% | 50% | 50% | 75% | 75% | 75% | 100% | 100% | 100% |
| **ART entry**  **(early)** | 0% | 0% | 0% | 0% | 0% | 0% | 0% | 0% | 0% | 0% | 0% | 0% | 0% |
| **Started ART** | 1,898,565 | 3,294,067 | 2,641,800 | 2,536,941 | 4,291,707 | 3,525,062 | 3,400,388 | 4,738,218 | 3,942,250 | 3,812,888 | 4,983,325 | 4,178,534 | 4,048,308 |
| **Total QALYs**  **(millions)** | 939 | 963 | 990 | 995 | 982 | 1,003 | 1,007 | 992 | 1,010 | 1,013 | 998 | 1,015 | 1,017 |
| **Total costs**  **($ billions)** | $282.2 | $292.1 | $334.5 | $340.2 | $299.8 | $343.5 | $349.0 | $303.7 | $348.0 | $353.7 | $306.0 | $350.8 | $356.5 |
| **Infections averted** |  | 845,113 | 3,297,323 | 3,625,333 | 1,512,972 | 3,545,397 | 3,826,716 | 1,839,917 | 3,673,717 | 3,930,471 | 2,030,990 | 3,751,962 | 3,993,700 |
| **CE ratio** |  | $421 | $1,029 | $1,050 | $413 | $958 | $992 | $410 | $929 | $967 | $408 | $914 | $954 |

| **ART Universal** | **Base case** | **(25 U,**  **0 Gen)** | **(25 U,**  **50 Gen)** | **(25 U,**  **100 Gen)** | **(50 U,**  **0 Gen)** | **(50 U,**  **50 Gen)** | **(50 U,**  **100 Gen)** | **(75 U,**  **0 Gen)** | **(75 U,**  **50 Gen)** | **(75 U,**  **100 Gen)** | **(100 U,**  **0 Gen)** | **(100 U,**  **50 Gen)** | **(100 U, 100 Gen)** |
| --- | --- | --- | --- | --- | --- | --- | --- | --- | --- | --- | --- | --- | --- |
| **Total Population** | 28,899,757 | 31,295,740 | 31,582,496 | 31,645,907 | 31,957,916 | 32,099,883 | 32,135,197 | 32,212,615 | 32,311,186 | 32,336,251 | 32,344,976 | 32,424,302 | 32,444,183 |
| **HIV population** | 3,010,186 | 2,521,362 | 1,957,002 | 1,885,503 | 2,429,248 | 2,020,691 | 1,965,384 | 2,392,339 | 2,038,629 | 1,991,250 | 2,372,222 | 2,045,369 | 2,002,584 |
| **HIV prevalence** | 10.4% | 8.1% | 6.2% | 6.0% | 7.6% | 6.3% | 6.1% | 7.4% | 6.3% | 6.2% | 7.3% | 6.3% | 6.2% |
| **PrEP entry** | 0% | 0% | 50% | 100% | 0% | 50% | 100% | 0% | 50% | 100% | 0% | 50% | 100% |
| **ART entry**  **(late & advanced)** | 10% | 25% | 25% | 25% | 50% | 50% | 50% | 75% | 75% | 75% | 100% | 100% | 100% |
| **ART entry**  **(early)** | 0% | 25% | 25% | 25% | 50% | 50% | 50% | 75% | 75% | 75% | 100% | 100% | 100% |
| **Started ART** | 1,898,565 | 4,966,719 | 4,223,726 | 4,089,185 | 5,605,789 | 4,997,687 | 4,883,560 | 5,833,240 | 5,292,816 | 5,194,604 | 5,946,284 | 5,442,264 | 5,354,623 |
| **Total QALYs**  **(millions)** | 939 | 1,006 | 1,017 | 1,019 | 1,025 | 1,031 | 1,033 | 1,033 | 1,038 | 1,039 | 1,037 | 1,041 | 1,042 |
| **Total costs**  **($ billions)** | $282.2 | $303.7 | $351.3 | $357.0 | $309.1 | $360.2 | $366.2 | $311.2 | $363.8 | $370.1 | $312.3 | $365.7 | $372.1 |
| **Infections averted** |  | 2,872,756 | 4,033,174 | 4,230,755 | 3,591,057 | 4,356,210 | 4,494,263 | 3,869,082 | 4,498,584 | 4,609,852 | 4,014,941 | 4,579,560 | 4,675,735 |
| **CE ratio** |  | $324 | $897 | $944 | $314 | $849 | $902 | $309 | $829 | $885 | $307 | $819 | $876 |

| **PrEP Focused** | **Base case** | **(10 G,**  **50 F)** | **(10 G,**  **100 F)** | **(25 G,**  **100 F)** | **(50 G,**  **100 F)** | **(75 G,**  **100 F)** | **(100 G, 100 F)** | **(25 U,**  **100 F)** | **(50 U,**  **100 F)** | **(75 U,**  **100 F)** | **(100 U, 100 F)** |
| --- | --- | --- | --- | --- | --- | --- | --- | --- | --- | --- | --- |
| **Total Population** | 28,899,757 | 29,582,098 | 30,075,564 | 30,708,858 | 31,218,742 | 31,473,542 | 31,624,528 | 31,647,941 | 32,147,612 | 32,349,957 | 32,457,488 |
| **HIV population** | 3,010,186 | 2,232,122 | 1,743,582 | 1,881,224 | 1,983,526 | 2,031,953 | 2,059,891 | 1,950,999 | 2,010,021 | 2,029,635 | 2,038,731 |
| **HIV prevalence** | 10.4% | 7.55% | 5.8% | 6.1% | 6.4% | 6.5% | 6.5% | 6.2% | 6.3% | 6.3% | 6.3% |
| **PrEP entry** | 0% | 50% | 100% | 100% | 100% | 100% | 100% | 100% | 100% | 100% | 100% |
| **ART entry**  **(late & advanced)** | 10% | 10% | 10% | 25% | 50% | 75% | 100% | 25% | 50% | 75% | 100% |
| **ART entry**  **(early)** | 0% | 0% | 0% | 0% | 0% | 0% | 0% | 25% | 50% | 75% | 100% |
| **Started ART** | 1,898,565 | 1,639,299 | 1,454,123 | 2,586,734 | 3,455,184 | 3,868,578 | 4,104,640 | 4,125,922 | 4,897,716 | 5,202,734 | 5,362,426 |
| **Total QALYs**  **(millions)** | 939 | 962 | 978 | 993 | 1,006 | 1,012 | 1,016 | 1,018 | 1,033 | 1,039 | 1,042 |
| **Total costs**  **($ billions)** | $282.2 | $277.1 | 274.9 | 284.7 | 292.9 | 297.2 | 299.8 | 299.7 | 307.9 | 311.4 | 313.3 |
| **Infections averted** |  | 1,837,744 | 3,084,508 | 3,393,762 | 3,642,543 | 3,766,653 | 3,840,111 | 4,160,422 | 4,468,827 | 4,595,331 | 4,663,411 |
| **CE ratio** |  | Cost saving | Cost saving | $47 | $163 | $206 | $229 | $222 | $276 | $293 | $302 |

| **PrEP General** | **Base case** | **(10 G, 25 Gen)** | **(10 G, 50 Gen)** | **(10 G, 75 Gen)** | **(10 G, 100 Gen)** |
| --- | --- | --- | --- | --- | --- |
| **Total Population** | 28,899,757 | 29,729,253 | 29,970,799 | 30,085,000 | 30,151,535 |
| **HIV population** | 3,010,186 | 1,882,906 | 1,693,209 | 1,618,892 | 1,579,445 |
| **HIV prevalence** | 10.4% | 6.33% | 5.65% | 5.38% | 5.24% |
| **PrEP entry** | 0% | 25% | 50% | 75% | 100% |
| **ART entry**  **(late & advanced)** | 10% | 10% | 10% | 10% | 10% |
| **ART entry**  **(early)** | 0% | 0% | 0% | 0% | 0% |
| **Started ART** | 1,898,565 | 1,574,594 | 1,485,240 | 1,443,919 | 1,420,126 |
| **Total QALYs**  **(millions)** | 939 | 968 | 975 | 978 | 980 |
| **Total costs**  **($ billions)** | $282.2 | $315.7 | $323.9 | $327.5 | $329.6 |
| **Infections averted** |  | 2,442,322 | 2,998,344 | 3,243,193 | 3,381,214 |
| **CE ratio** |  | $1,187 | $1,172 | $1,163 | $1,158 |

**Additional file 1 Table 3**: Outcomes over 20 years of various strategies to scale up single or combination HIV prevention and treatment programs: Guidelines ART (individuals with CD4 cell counts ≤ 350 cells/μl only), Universal ART (all HIV infected individuals), General Pre-exposure prophylaxis (general population), Focused Pre-exposure prophylaxis (individuals at high-risk of acquiring HIV).

Program name abbreviation key: G=Guidelines ART, U= Universal ART; Gen=General PrEP, F=Focused PrEP

**Additional file 1 Figure 1:** Model diagram


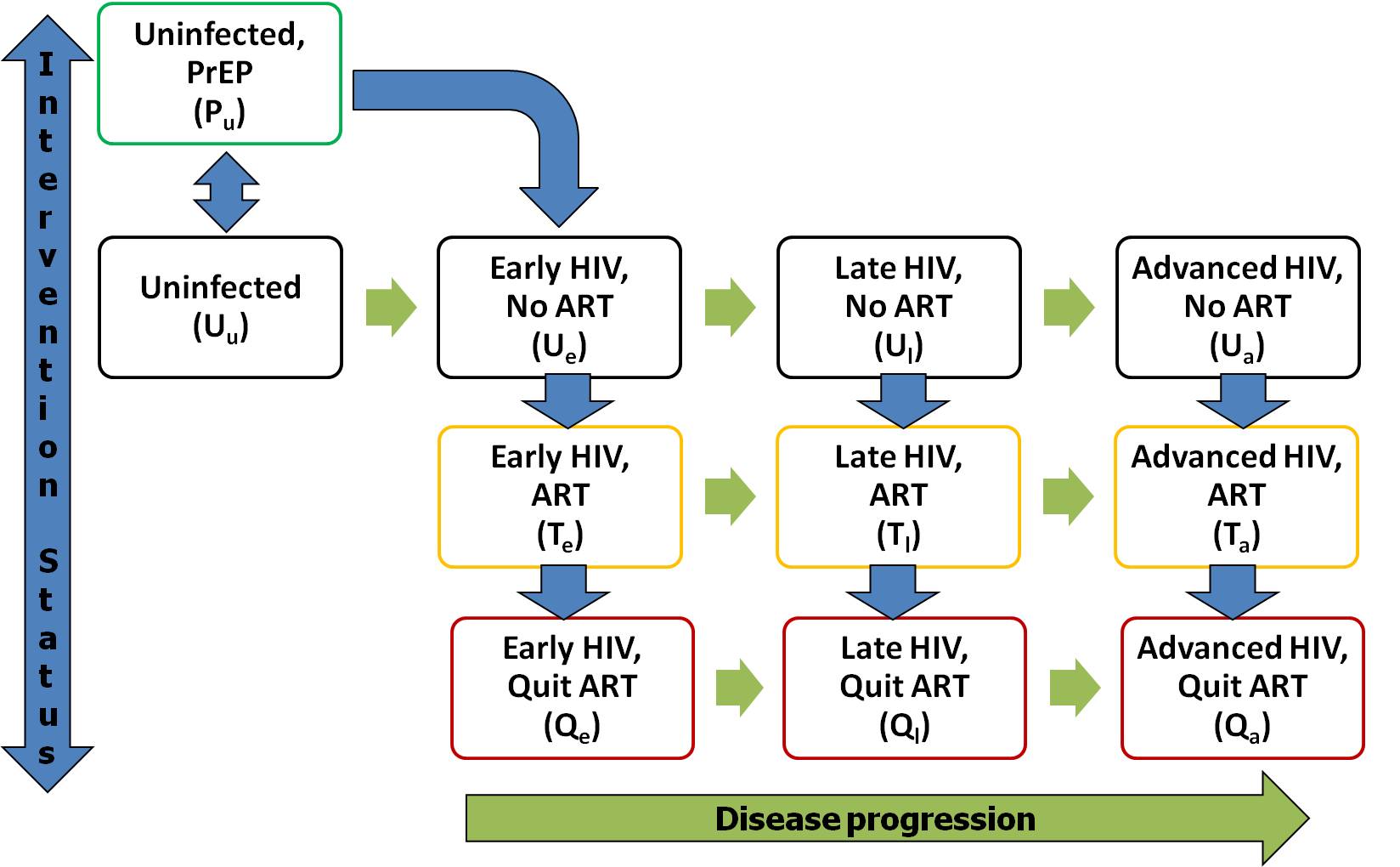


**Additional file 1 Figure 2**:

Infections averted over 20 years with various levels of scale up for single or combination programs: Guidelines ART (individuals with CD4 cell counts ≤ 350 cells/μl only), Universal ART (all HIV infected individuals), General Pre-exposure prophylaxis (general population), Focused Pre-exposure prophylaxis (individuals at high-risk of acquiring HIV). The strategies are presented in order of increasing costs, ranging from $275-$375 billion. The bar colors indicate type of ART program, the bar outline indicates type of PrEP program.

ART strategies indicated by bar color: Guidelines – Light gray; Universal – Dark gray

PrEP strategies indicated by bar outline: General - Solid line; Focused - Dashed line


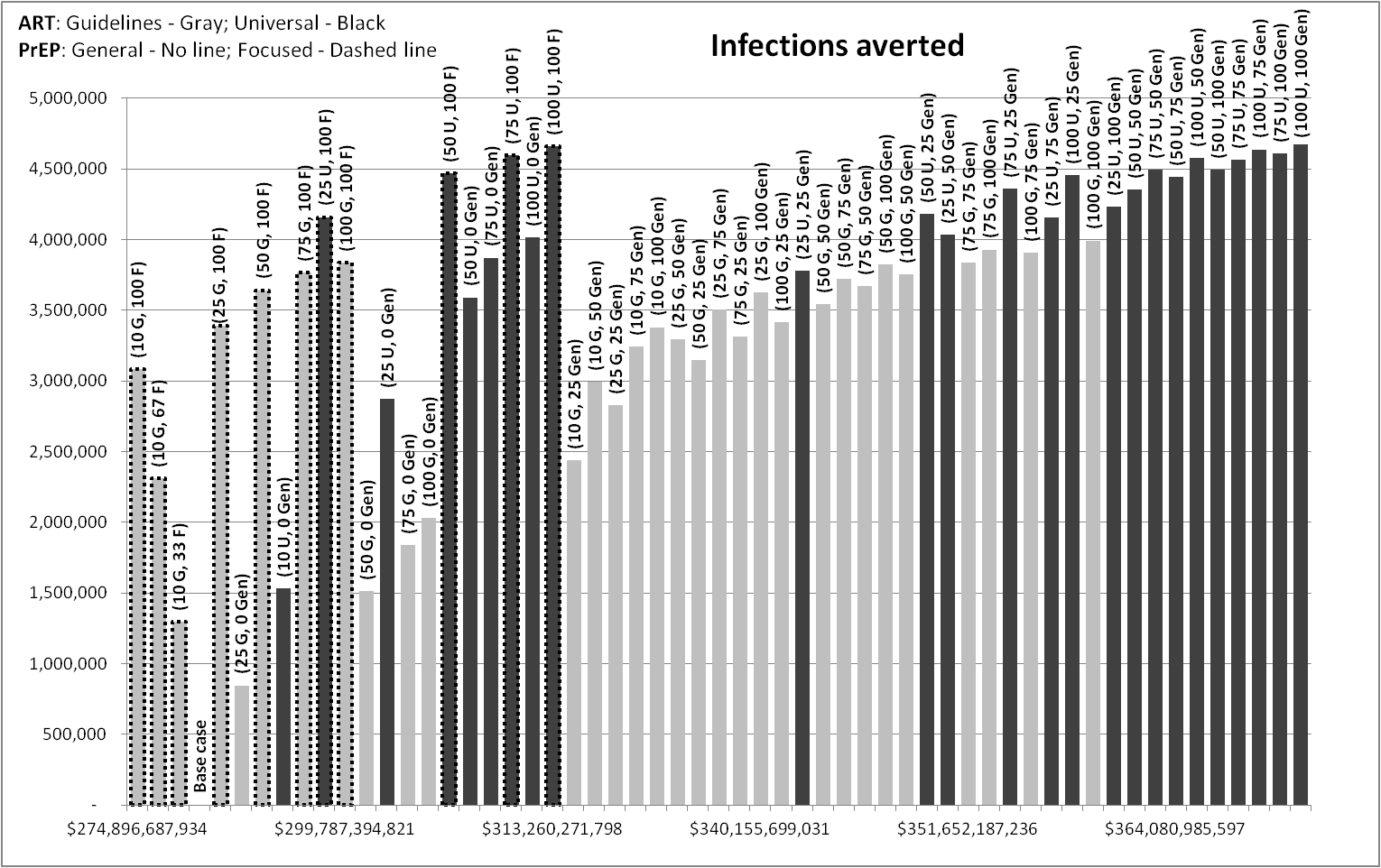


**References**

1. Shisana O, Rehle T, Simbayi L, Zuma K, Jooste S, Pillay-van-Wyk V, Mbelle N, Van Zyl J, Parker W, Zungu N *et al*: **South African national HIV prevalence, incidence, behaviour and communication survey 2008: A turning tide among teenagers?** In*.* Cape Town, South Africa: Human Sciences Research Council; 2009.

2. Pinkerton SD, Abramson PR: **Effectiveness of condoms in preventing HIV transmission**. *Social Science & Medicine* 1997, **44**(9):1303-1312.

3. Cohen MS, Chen YQ, McCauley M, Gamble T, Hosseinipour MC, Kumarasamy N, Hakim JG, Kumwenda J, Grinsztejn B, Pilotto JHS *et al*: **Prevention of HIV-1 Infection with Early Antiretroviral Therapy**. *New England Journal of Medicine* 2011, **365**(6):493-505.

4. Baeten J, Celum C: **Antiretroviral preexposure prophylaxis for HIV-1 prevention among heterosexual African men and women: the Partners PrEP Study.** In: *6th IAS Conference on HIV Pathogenesis, Treatment and Prevention.* Rome, Italy; 2011.

5. Thigpen MC, Kebaabetswe PM, Smith DK, Segolodi TM, Soud FA, Chillag K, Chirwa LI, Kasonde M, Mutanhaurwa R, Henderson FL *et al*: **Daily oral antiretroviral use for the prevention of HIV infection in heterosexually active young adults in Botswana: results from the TDF2 study**. In: *6th International AIDS Society Conference on HIV Pathogenesis, Treatment and Prevention.* Rome, Italy; 2011.

6. Joint United Nations Programme on HIV/AIDS (UNAIDS): **Global report: UNAIDS report on the global AIDS epidemic 2010**. In*.* Geneva, Switzerland; 2010.

7. World Health Organization (WHO): **Global Health Observatory Data Repository: South Africa Life Tables**. In*.*; 2012.

8. Badri M, Lawn SD, Wood R: **Short-term risk of AIDS or death in people infected with HIV-1 before antiretroviral therapy in South Africa: a longitudinal study**. *The Lancet* 2006, **368**(9543):1254-1259.

9. Badri M, Maartens G, Mandalia S, Bekker L-G, Penrod JR, Platt RW, Wood R, Beck EJ: **Cost-Effectiveness of Highly Active Antiretroviral Therapy in South Africa**. *PLoS Med* 2005, **3**(1):e4.

10. Hollingsworth TD, Anderson RM, Fraser C: **HIV-1 Transmission, by Stage of Infection**. *Journal of Infectious Diseases* 2008, **198**(5):687-693.

11. Tengs TO, Lin TH: **A Meta-Analysis of Utility Estimates for HIV/AIDS**. *Medical Decision Making* 2002, **22**(6):475-481.

12. Cleary S, McIntyre D, Boulle A: **The cost-effectiveness of Antiretroviral Treatment in Khayelitsha, South Africa - a primary data analysis**. *Cost-Effectiveness and Resource Allocation* 2006, **4**(1):20.

13. Medecins Sans Frontieres (MSF): **Untangling the web of antiretroviral price reductions**. In*.*; 2011.

14. Motsoaledi A: **Massive reduction in ARV prices**. In*.* Cape Town, South Africa; 2010.
